# Supplementary material for: Radiocesium concentrations in mushrooms collected in Kawauchi Village five to eight years after the Fukushima Daiichi Nuclear Power Plant accident
Source: PLoS One. 2020 Sep 15;15(9):e0239296. doi: 10.1371/journal.pone.0239296 (PMC7491737; doi:10.1371/journal.pone.0239296)
Supplement: S2 File — (DOCX) [file pone.0239296.s002.docx]

**S1 Table. Activity concentrations of radiocesium (Bq/kg) in mushroom samples (27 species) collected in Kawauchi village from 2016 to 2019.**

| Species | Sample year | n | ^134^Cs (Bq/kg) | ^137^Cs (Bq/kg) |
| --- | --- | --- | --- | --- |
|  |  |  | Median (Min-Max) * | Median (Min-Max) |
| Hygrophorus russula | 2016 | 12 | 230 (113–468) | 1293 (694–2,666) |
|  | 2017 | 1 | 155 | 1177 |
|  | 2018 | 4 | 112 (42–173) | 1,150 (423–1,864) |
| Boletopsis leucomelas | 2016 | 7 | 98 (86–335) | 600 (455–2,213) |
|  | 2018 | 4 | 41 (29–78) | 490 (232–878) |
|  | 2019 | 6 | 19 (n.d.*–55) | 363 (120–892) |
| Hypholoma sublateritium | 2016 | 2 | 55 (52–58) | 371 (333–409) |
|  | 2018 | 2 | 11 (n.d.–12) | 74 (18–131) |
|  | 2019 | 3 | 14 (n.d.–17) | 213 (30–223) |
| Tricholoma equestre | 2016 | 2 | 31 (21–40) | 182 (15–19) |
|  | 2018 | 1 | n.d. | 41 |
| Tricholoma ustale | 2016 | 2 | 51 (14–88) | 300 (121–478) |
|  | 2018 | 1 | 17 | 194 |
| Cortinarius caperatus | 2016 | 2 | 440 (12–868) | 2485 (57–4,913) |
|  | 2018 | 1 | 182 | 2017 |
| Tricholoma matsutake | 2016 | 2 | 53 (20–86) | 316 (117–514) |
|  | 2019 | 4 | n.d. (n.d.–12) | 101 (90–115) |
| Tricholoma portentosum | 2018 | 1 | 100 | 1,060 |
|  | 2019 | 1 | n.d. | 145 |
| Lyophyllum decastes | 2016 | 7 | n.d. (n.d.–24) | 11 (n.d.–106) |
|  | 2017 | 2 | n.d. | n.d.–10 |
|  | 2018 | 1 | n.d. | n.d. |
| Grifola frondosa | 2016 | 3 | n.d. (n.d.–25) | 69 (8–181) |
|  | 2018 | 2 | 41 (35–47) | 487 (379–595) |
| Lepista nuda | 2016 | 1 | n.d. | 14 |
|  | 2018 | 1 | 537 | 5733 |
| Armillaria mellea | 2016 | 1 | n.d. | n.d. |
|  | 2018 | 1 | 17 | 190 |
| Auricularia auricula–judae | 2016 | 1 | 18 | 178 |
| Cortinarius salor Fr. | 2016 | 4 | 510 (445–1488) | 2925 (2,473–8451) |
| Lactarius volemus | 2016 | 3 | 24 (n.d.–26) | 118 (23–161) |
| Tricholoma terreum | 2016 | 2 | n.d. | 18 (n.d.–36) |
| Lactarius hatsudake | 2016 | 1 | n.d. | 224 |
| Pholiota squarrosa | 2016 | 4 | n.d | 12 (n.d.–20) |
| Leucopaxillus giganteus | 2016 | 3 | n.d | 28 (9–31) |
| Lentinula edodes | 2016 | 2 | 62 (26–97) | 367 (185–549) |
| Pleurotus ostreatus | 2016 | 1 | 25 | 148 |
| Entoloma sinuatum | 2016 | 1 | 48 | 331 |
| Entoloma sarcopum | 2018 | 2 | 22（14–30） | 267（141–394） |
| Cortinarius tenujpes | 2018 | 3 | 15 (14–21) | 169 (153–231) |
| Pholiota microspora | 2018 | 1 | 151 | 1578 |
| Ramaria botrytis | 2019 | 3 | 25（14–34） | 317.1（268–537） |
| Clitocybe | 2019 | 2 | 35 (n.d.–59) | 453 (32–875） |

*Min: minimum; Max: maximum; n.d.: could not be determined.
